# Supplementary material for: miR-302 Suppresses the Proliferation, Migration, and Invasion of Breast Cancer Cells by Downregulating ATAD2
Source: Cancers (Basel). 2022 Sep 6;14(18):4345. doi: 10.3390/cancers14184345 (PMC9497224; doi:10.3390/cancers14184345)

Figure S1

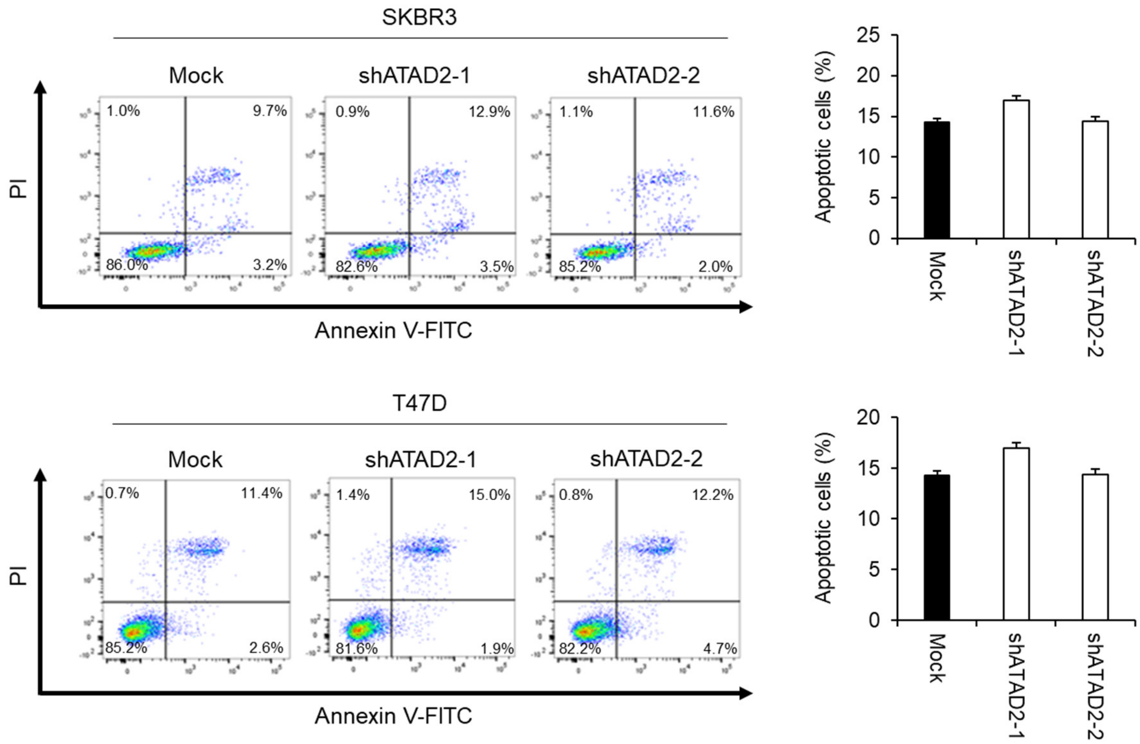

Supplementary Figure S1. The ATAD2 depletion does not affect the apoptosis in SKBR3 and T47D breast cancer cell lines. Indicated cells were seeded into a 6-well plate. After 3 days, Cells were stained with annexin-V FITC and propidium iodide. Apoptotic cells were analyzed by FACS.

Figure S2

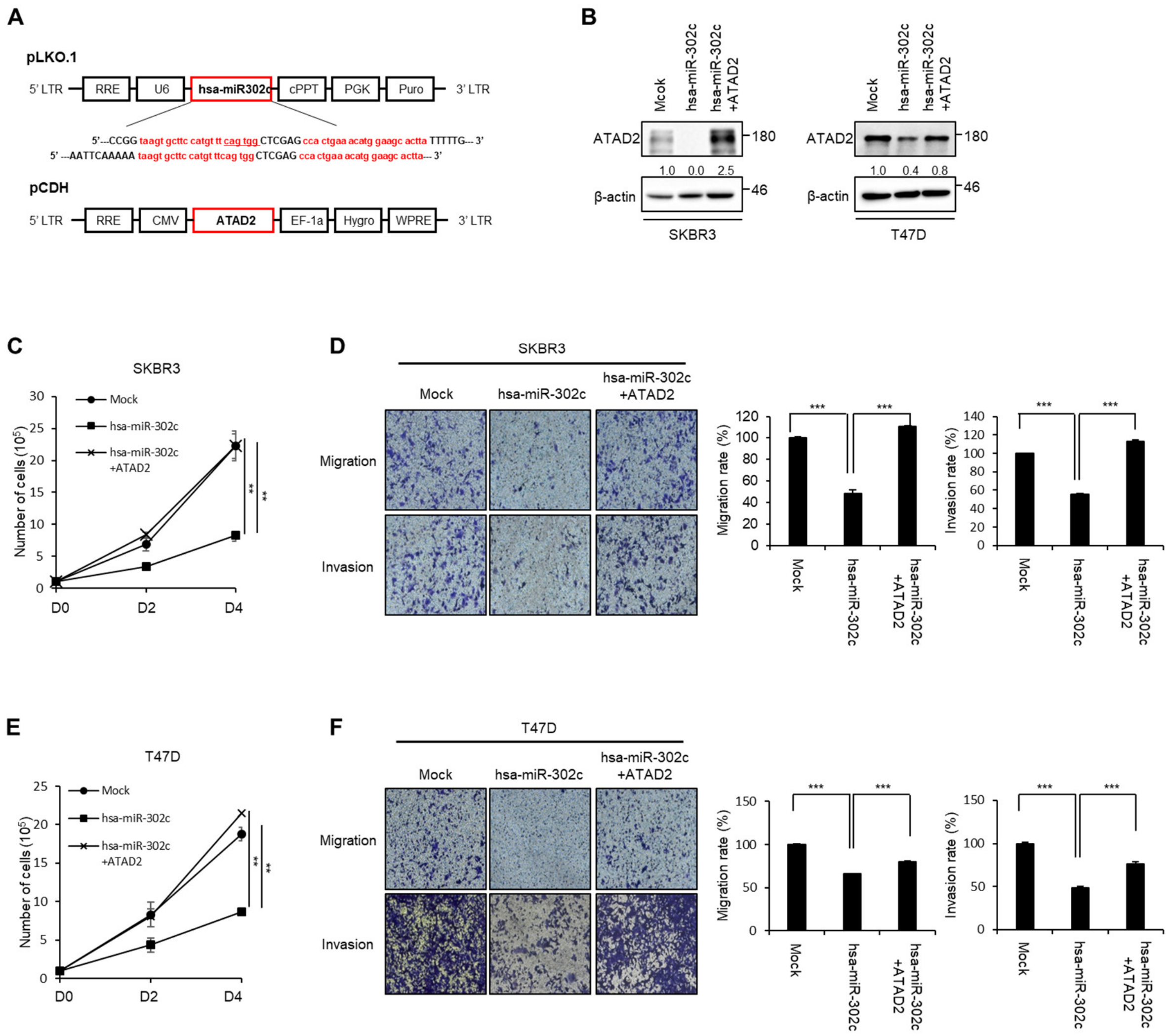

Supplementary Figure S2. Restoration of ATAD2 rescued the inhibition of proliferation, migration, and invasion ability caused by miR-302c. (A) miR-302c and ATAD2 expression vector constructs (B) ATAD2 expression levels in Mock, miR-302, and miR-302/ATAD2 overexpressing SKBR3 and T47D cells. (C and E) Proliferation rates of indicated cells were calculated as described in Materials and Methods. (D and F) Migration and invasion abilities were analyzed as described in Materials and Methods. Graphs represent migration and invasion rates (%) by crystal violet staining. Statistical analyses were performed using paired two-tailed Student's t-test. * $p < 0.05$, ** $p < 0.01$.

Figure S3. Uncropped blots

Fig1. E

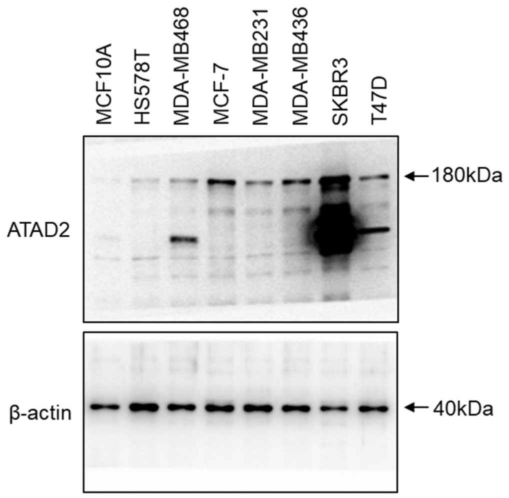

Fig2. A

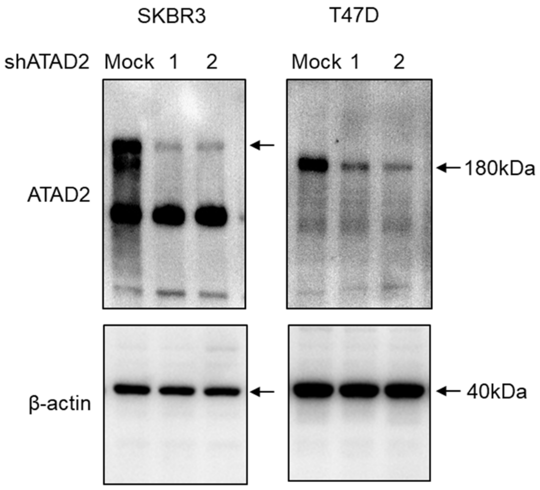

Fig3. B

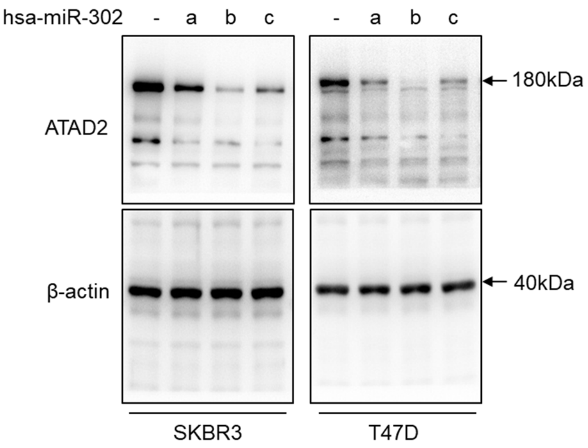

Fig5. A

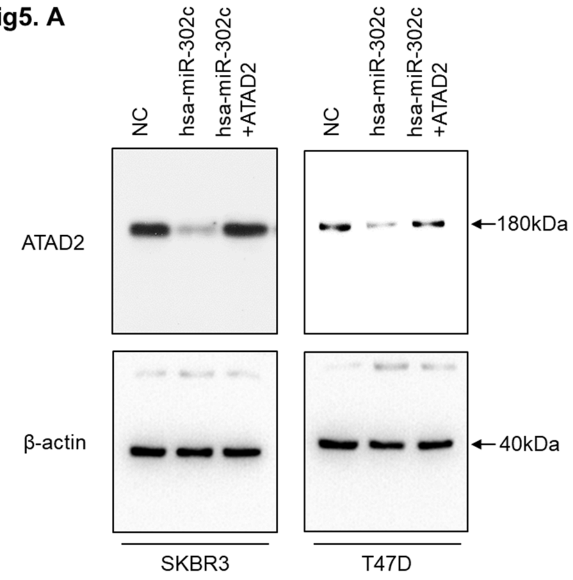

Figure S3. Uncropped blots (continued)

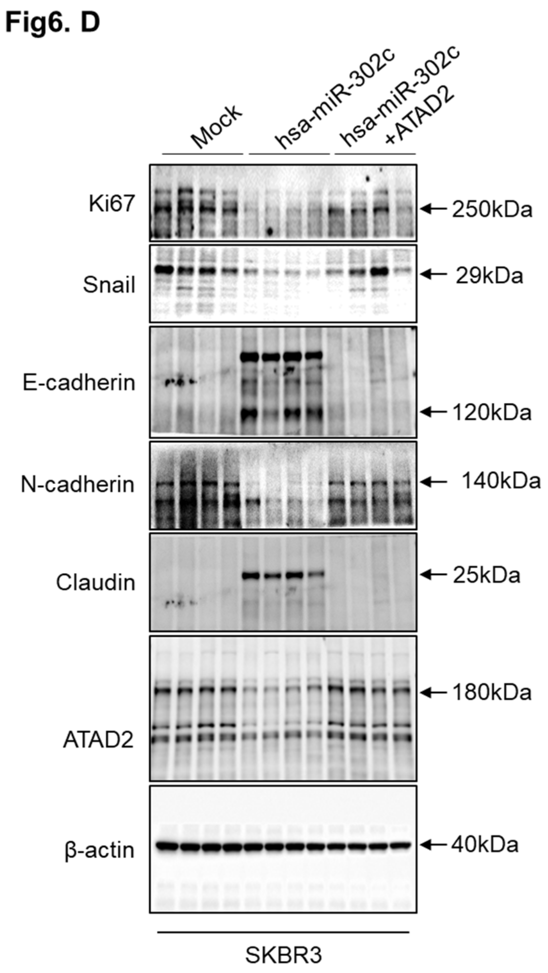

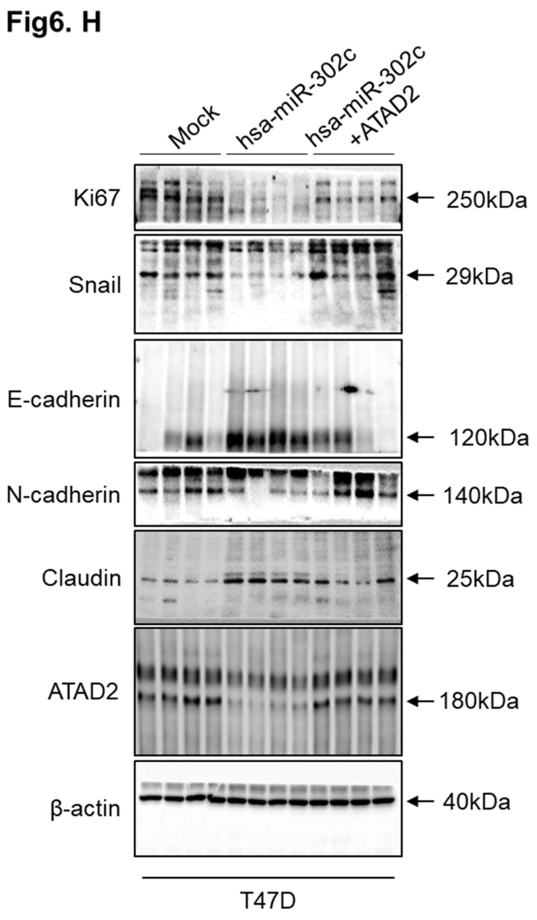

Supplement: Supplementary file 1 [file cancers-14-04345-s001.zip › cancers-1864835-supplementary.pdf]
